# Perspectives on acute myeloid leukemia diagnosis: a comparative analysis of the latest World Health Organization and the International Consensus Classifications

**DOI:** 10.1038/s41375-023-01996-9

**Published:** 2023-08-14

**Authors:** Jin Jung, Daehun Kwag, Yonggoo Kim, Jong-Mi Lee, Ari Ahn, Hoon Seok Kim, Byunggyu Bae, Silvia Park, Hee-Je Kim, Byung-Sik Cho, Myungshin Kim

**Affiliations:** 1 Department of Laboratory Medicine, College of Medicine, The Catholic University of Korea, Seoul, Republic of Korea; 2 Catholic Genetic Laboratory Center, Seoul St. Mary’s Hospital, College of Medicine, The Catholic University of Korea, Seoul, Republic of Korea; 3 Department of Hematology, Catholic Hematology Hospital, Seoul St. Mary’s Hospital, College of Medicine, The Catholic University of Korea, Seoul, Republic of Korea

**Keywords:** Acute myeloid leukaemia, Myelodysplastic syndrome

## To the Editor

Recently, the 5th edition of ‘The World Health Organization (WHO) Classification of Tumors of Haematopoietic and Lymphoid Tissues’ (WHO2022) was released in beta version [1]. In WHO2022, the classification of AML underwent changes, separating AML with defining genetic abnormalities from AML defined by differentiation (AML-Diff). Additionally, AML with myelodysplasia-related changes (AML-MRC) was renamed ‘AML myelodysplasia-related’ (AML-MR), with updates including the removal of morphology as a sole diagnostic premise, revised cytogenetic criteria, and a mutation-based definition. An independent proposal, the International Consensus Classification (ICC), was also published during the same period [2]. This study aims to compare and analyze these two classifications, focusing on AML’s diagnostic criteria and entity definition.

## Methods

### Patients

**Fig. 1:**
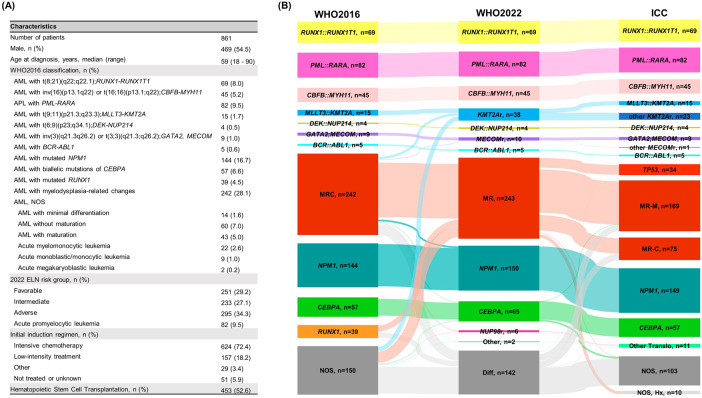
Baseline characteristics and diagnosis comparisom by classification. **A** Baseline characteristics of patients in this study. **B** Changes in acute myeloid leukemia (AML) diagnoses according to WHO2022 and ICC. WHO World Health Organization classification, NOS not otherwise specified, ELN European LeukemiaNet, ICC International Consensus Classification, MRC acute myeloid leukemia with myelodysplasia-related changes, KMT2Ar KMT2A rearrangement, MECOMr MECOM rearrangement, MR acute myeloid leukemia with myelodysplasia-related, NUP98r NUP98 rearrangement, Other acute myeloid leukemia with other defined genetic alterations, Diff acute myeloid leukemia, defined by differentiation, MR-M acute myeloid leukemia with myelodysplasia-related gene mutations, MR-C acute myeloid leukemia with myelodysplasia-related cytogenetic abnormalities, Other translo acute myeloid leukemia with other rare recurring translocations, Hx with history of myelodysplastic syndrome or myelodysplastic syndrome/myeloproliferative disorder.

A total of 861 newly-diagnosed AML patients aged ≥18 years, according to the revised 4th WHO classification (WHO2016) [3], were included from Oct. 2017 and Oct. 2021 at Seoul St. Mary’s Hospital, College of Medicine, The Catholic University of Korea (Fig. 1A). Bone marrow samples were independently reviewed and re-classified by five experienced haematopathologists (JJ, YK, J-ML, AA and MK). Risk stratification followed the 2022 European LeukemiaNet (ELN) classification [4]. The study’s last follow-up was Dec. 22nd, 2022 for survivors. Institutional Review Board approval was obtained (IRB No: KC23RISI0243).

Chi-square, Fisher’s exact, Mann–Whitney U, and Kruskal–Wallis H tests were used for comparison. Kaplan–Meier analysis with log-rank test was applied to plot overall survival (OS) curves. Prism version 9.5.1 for Windows (GraphPad, San Diego, CA, USA) and MedCalc 20.121 (MedCalc Software, Ostend, West-Vlaanderen, Belgium) were used.

## Results

### Reclassification of AML according to WHO2022 and ICC

There was no change in the classification for 205 patients with defining genetic abnormalities (*RUNX1::RUNX1T1*, *PML::RARA*, *CBFB::MYH11*, *DEK::NUP214* and *BCR::ABL1* fusions) between WHO2016 and WHO2022 (Fig. 1B). Under the WHO2022, which encompasses any partner gene rearranged with *KMT2A* in ‘AML with *KMT2A* rearrangement’, an additional 23 patients were reclassified into this category (Supplementary Table 1). These patients had *KMT2A* rearrangements involving genes other than *MLLT3* and were originally diagnosed as AML-MRC (*n* = 8) and AML not otherwise specified (AML-NOS) (*n* = 15). A total of 14 fusion partners were detected, with *AFDN* (7.9%, *n* = 3), *SEPT9* (7.9%, *n* = 3), and *ELL* (7.9%, *n* = 3) being the most frequently observed. Furthermore, updated classification introduced two new categories: *NUP98* rearrangement (6 patients) and other genetic alterations (2 patients). The number of patients diagnosed as ‘AML with *CEBPA* mutation’, including both biallelic mutations and single mutations located in the basic leucine zipper (bZIP) region, was increased to 65 (7.5%) according to WHO2022, representing an additional 8 patients compared to 57 (6.6%) who were diagnosed as ‘AML with biallelic mutations of *CEBPA*’ according to WHO2016. AML-MR employed significantly new essential diagnostic criteria in WHO2022. A total of 243 patients were diagnosed as AML-MR. Majority (*n* = 186, 76.5%) of them were included in AML-MRC by WHO2016 while a considerable proportion were previously classified as ‘AML with *RUNX1* mutation’ (*n* = 20, 8.2%) or AML-NOS (*n* = 37, 15.2%) by the same system. The *DDX41* germline mutation, the most common genetic predisposition to MDS and AML, was identified in 31 patients, including 27 with two mutations (germline and somatic), and 4 with a single mutation. We found an additional 4 patients with a single somatic *DDX41* mutation.

Comparing to WHO2022, the ICC classified *NUP98* rearrangements as “other rare recurring translocations” and two gene fusions (*PRDM16::RPN1* and *RUNX1::CBFA2T3*) were classified as ‘other rare recurring translocations’, whereas the WHO2022 classified them as AML-Diff (Supplementary Table 2). ICC implemented three categories: AML with mutated *TP53* (AML-TP53), AML with myelodysplasia-related gene mutations (AML-MR-M), and cytogenetic abnormalities (AML-MR-C) (Supplementary Table 3). Thirty-four patients were diagnosed with AML-TP53. Most belonged to AML-MR, except for one in AML-Diff according to WHO2022. Within AML-TP53, 6 patients had multiple mutations, and 9 had a single mutation along with allele deletion, and 12 showed a *TP53* mutation with variant allele fraction >49%, suggesting combined copy loss [5]. A total of 169 patients were diagnosed with AML-MR-M, and 75 patients as AML-MR-C by ICC. ICC criteria did not incorporate the ‘history of myelodysplastic neoplasm (MDS) or myelodysplastic/myeloproliferative neoplasm (MDS/MPN)’ as a criterion for categorizing AML-MR. As a result, 10 patients who were classified as AML-MR by WHO2022 were assigned to the AML-NOS category by ICC.

### Gene profile in AML

**Fig. 2:**
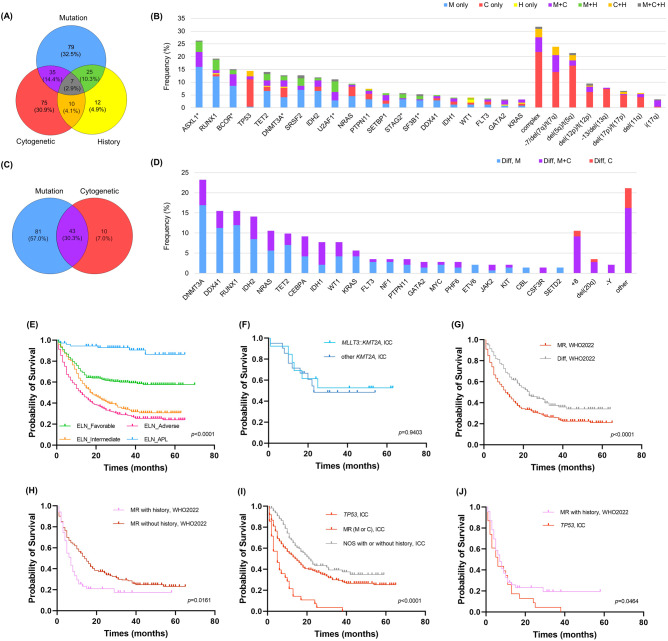
Characterization of patients with acute myeloid leukemia, myelodysplasia-related (AML-MR), and acute myeloid leukemia defined by differentiation (AML-Diff) according to WHO2022, and overall survival of acute myeloid leukemia (AML) in this study. **A** Venn diagram depicting overlap and number (proportion) across three groups of AML-MR according to the WHO2022 classification. **B** The frequency of 20 mutated genes and myelodysplasia-related cytogenetic abnormalities (>3%) in AML-MR patients according to the WHO2022 classification. *mutated gene defining AML with myelodysplasia-related. **C** Venn diagram depicting the overlap and number (proportion) between differences of genetic abnormalities in AML-Diff according to the WHO2022 classification. **D** The frequency of 22 mutated genes and cytogenetic abnormalities (>1%) in AML-Diff patient according to the WHO2022 classification. **E** Overall survival of AML according to 2022 ELN risk classification. **F** Overall survival of *KMT2A* rearrangement AML according to ICC (**G**), (**H**) Overall survival of AML-MR according to WHO2022. **I** Overall survival of AML-TP53, AML-MR with myelodysplasia-related gene mutations or cytogenetic abnormalities, and AML-NOS according to ICC. **J** Overall survival of AML-MR with history according to WHO2022 and AML-TP53 according to ICC. Significance was tested by Kaplan–Meier survival analysis. WHO World Health Organization, M only AML-MR with only mutations, C only AML-MR with only cytogenetic abnormalities, H only AML-MR with only a history of myelodysplastic myelodysplastic syndrome (MDS) or myelodysplastic syndrome/myeloproliferative disorder (MDS/MPN), M + C AML-MR with both mutations and cytogenetic abnormalities, M + H AML-MR with both mutations and a history of MDS or MDS/MPN, C + H AML-MR with both cytogenetic abnormalities and a history of MDS or MDS/MPN, M + C + H AML-MR fulfilled all three criteria, Complex Complex karyotype (≥3 abnormalities), t(5p) loss of 5q due to unbalanced translocation, t(7q) loss of 7q due to unbalanced translocation, t(12p) loss of 12p due to unbalanced translocation, t(17p) loss of 17p due to unbalanced translocation, Diff, M AML-Diff with only mutations, Diff, M + C AML-Diff with both mutations and cytogenetic abnormalities, Diff, C AML-Diff with only cytogenetic abnormalities, ELN European LeukemiaNet, ICC International Consensus Classification, AML-TP53 AML with mutated TP53, history history of myelodysplastic syndrome or myelodysplastic syndrome/myeloproliferative disorder.

Of 243 AML-MR patients classified by WHO2022, 75 had only cytogenetic abnormalities, 79 had only mutations, and 12 had only a history of MDS or MDS/MPN, while 77 patients fulfilled at least two of the essential diagnostic criteria and seven patients fulfilled all three criteria (Fig. 2A, Supplementary Fig. 1). Regarding cytogenetic abnormalities, complex karyotype was the most frequently detected (31.7%), followed by −7/del(7q) (23.9%), and del(5q) (21.4%) (Fig. 2B). Regarding mutations, 222 (89.2%) patients had at least one mutation, including 53 (21.3%) patients with one mutation, 64 (25.7%) patients with two mutations, and 105 (42.2%) patients with three or more mutations. *ASXL1* was the most frequently mutated one (26.3%), followed by *RUNX1* (19.3%), *BCOR* (15.2%), *TP53* (14.4%), *TET2*(14.0%), *DMNT3A* (12.8%), *SRSF2* (12.8%), *IDH2* (11.9%), and *U2AF1* (11.1%).

Among the 142 AML-Diff patients diagnosed by WHO2022, 124 had molecular mutations, 53 showed cytogenetic abnormalities (Fig. 2C), and 43 patients had both genetic mutations and cytogenetic abnormalities. Trisomy 8 was most common (10.6%), followed by del(20q) (3.5%) and -Y (2.1%) (Fig. 2D, Supplementary Fig. 2). ICC includes +8 and del(20q) as an additional cytogenetic abnormality in the classification of AML-MR-C, resulting in the classification of additional 19 patients under this category. In terms of mutations, *DMNT3A* (23.2%) was the most frequently mutated gene, followed by *DDX41* (15.5%), *RUNX1* (15.5%), *IDH2* (14.1%), and *NRAS* (10.6%). The ICC includes *RUNX1* mutation as an additional molecular abnormality in the classification of AML-MR-M, resulting in the classification of 22 patients under this category. Only eight patients of AML-Diff did not possess any genetic abnormalities.

### Clinical outcomes

Median follow-up duration was 17 months (95% CI: 15.3–20.0 months). Three-year OS was 42.5% (95% CI: 39.0–45.9%). The prognostic accuracy of the 2022 ELN criteria was demonstrated in our dataset (Fig. 2E). No significant difference in OS was observed when comparing ‘AML with *KMT2A::MLLT3*’ and ‘AML with *KMT2A* rearrangement other than *KMT2A::MLLT3*’ (Fig. 2F, Supplementary Table 4). Patients with AML-MR in the WHO2022 had significantly shorter survivals than those with AML-Diff (10.0 months [95% CI: 7.0–13.0] vs. 23.0 months [95% CI: 17.0–31.0], *p* < 0.0001) (Fig. 2G). AML-Diff subgroups in the WHO2022 did not present a statistically significant difference in OS. When examining AML-MR subgroup, patients with history of MDS or MDS/MPN had shorter survival than those without the history (6.0 months [95% CI: 4.0–8.0] vs. 13.0 months [95% CI: 9.0–16.0], *p* = 0.0161) (Fig. 2H). According to the ICC criteria, AML-TP53 showed the shortest OS, followed by AML-MR (M or C) and AML-NOS (3.0 months [95% CI: 2.0–6.0] vs. 13.0 months [95% CI: 10.0–17.0] vs. 21.0 months [95% CI: 15.0–31.0], *p* < 0.0001) (Fig. 2I). In addition, AML-TP53 by ICC had shorter OS than those with AML-MR subgroup with history of MDS or MDS/MPN by WHO2022 (*p* = 0.0464) (Fig. 2J).

## Discussion

Using WHO2022, 154 patients were reclassified from WHO2016, including 23 with *KMT2A* rearrangement and an additional 23 with other genetic abnormalities [6]. The WHO2022 had a significant impact on the AML-MR category. The majority of cases were originally classified as AML-MRC according to WHO2016, while 8.2% were reclassified from ‘AML with *RUNX1* mutation’ and 15.2% from AML-NOS. The redefined AML-MR appears to provide a clear and simplified diagnostic approach as it removes morphology alone as a diagnostic criterion [7, 8]. Further, the AML-MR patients exhibited significantly worse survival outcomes compared to AML-Diff patients. In terms of genetics, all AML-MR had genetic abnormalities, particularly those associated with adverse risk group [9]. Among AML-Diff patients, 88.8% had genetic abnormalities falling into the favorable or intermediate risk groups [4]. Although there were some differences in defining AML-MR in ICC, the importance of clarifying the diagnostic criteria was not diminished. AML-MR (M or C) by ICC showed a worse clinical outcome compared to AML-NOS. While further research is required to better comprehend the relationships between these genetic aberrations with disease development/pathogenesis, it is evident that patients diagnosed with AML-MR based on the WHO2022 benefit from improved criteria.

This study, along with previous studies, has demonstrated that patients with history of MDS or MDS/MPN had a poor prognosis, likely due to the failure of hypomethylating agent treatment [10–12]. Similarly, AML-TP53, as indicated in the ICC, is associated with the poorest prognosis [13], emphasizing the importance of identifying these patients. In our study, we found that due to the minimal overlap between these two groups (only 5 patients), it is crucial to consider both classifications independently for risk stratification. Another noteworthy point is the increasing reliance on molecular techniques such as NGS, which may not be readily accessible in many hospitals. While these classifications are crucial for patient care [14], it is imperative to establish NGS as a part of routine practice to ensure the best possible care for patients.

In conclusion, our evaluation supports the refinements made in the WHO2022 classification for AML, and additionally incorporates the recommendations from ICC. Clinical, hematopathological, and genetic characteristics accumulated over the past two decades have contributed to the refinement of these classifications and the identification of new entities.

### Supplementary information

Supplemental infromation
