# Supplementary material for: Perspectives on acute myeloid leukemia diagnosis: a comparative analysis of the latest World Health Organization and the International Consensus Classifications
Source: Leukemia. 2023 Aug 14;37(10):2125–8. doi: 10.1038/s41375-023-01996-9 (PMC10539164; doi:10.1038/s41375-023-01996-9)
Supplement: Supplementary file 1 — Supplemental infromation [file 41375_2023_1996_MOESM1_ESM.docx]

**Supplemental Information**

**Supplementary Methods**

**Supplementary Table1.** Comparison of the distribution of acute myeloid leukemia patients between WHO 2022 and ICC classification

**Supplementary Table2.** Comparison of the distribution of acute myeloid leukemia patients between WHO 2016 and WHO 2022 classifications

**Supplementary Table3.** Comparison of the distribution of acute myeloid leukemia patients between WHO 2016 and ICC classification

**Supplementary Table4.** Overall survival of acute myeloid leukemia subgroups

**Supplementary Figure1.** Genetic profile of acute myeloid leukemia with myelodysplasia-related (AML-MR) patients according to WHO 2022

**Supplementary Figure2.** Genetic profile of acute myeloid leukemia, defined by differentiation (AML, diff) patients according to WHO 2022

**Supplementary Methods:**

**Multi-parameter flow cytometry**

Immunophenotyping of leukemic cells was performed using flow cytometry with selected combinations of four antibodies conjugated to the following fluorochromes: fluorescein isothiocyanate (FITC), phycoerythrin (PE), peridinin chlorophyll protein–cyanin 5.5 (PerCPCy5.5), and allophycocyanin (APC) (Becton Dickinson, San Jose, CA, USA). The following antibodies were used: cytoplasmic myeloperoxidase (cyMPO), cyCD3, cyCD79a, CD13, CD33, CD34, CD11c, CD64, CD117, CD10, CD19, CD2, CD4, CD5, CD7, CD8, HLA-DR, and CD45 (Becton Dickinson). At least 10,000 events were acquired. Leukemic cells were analyzed using a BD FACS Canto II instrument with DIVA software (Becton Dickinson).

**Cytogenetic analyses**

Chromosomal analyses were performed by examining short-term cultures of BM specimens according to standard conventional cytogenetic protocols. At least 20 cells in metaphase were analyzed for each case. Clonal abnormalities were classified according to the 2020 International System for Human Cytogenetic Nomenclature guidelines (1).

**Molecular analyses**

Next-generation sequencing was performed using initial BM aspirates and St. Mary’s customized NGS panel for acute leukemia ('SM Acute leukemia panel') covering 67 genes (2). Template was prepared with an Ion Chef™ System. Sequencing was performed with an Ion S5 XL Sequencer (Thermo Fisher Scientific, Waltham, MA, USA) according to the manufacturer’s instructions. Sequenced reads were mapped to the human reference genome (hg19, Genome Reference Consortium, February 2009). Variants with more than 20 reads and 5% variant allele frequencies (VAFs) were considered to be mutated. Annotated variants were classified into four tiers according to the Standards and Guidelines of the Association for Molecular Pathology (3). *FLT3*-internal tandem duplication mutations were also analyzed using fragment analysis as described previously (4).

Supplementary Table1. Comparison of the distribution of acute myeloid leukemia patients between WHO2016 and WHO2022 classifications

| WHO2022 | | *RUNX1::* *RUNX1T1* | *PML::*  *RARA* | *CBFB::*  *MYH11* | *KMT2A* | *DEK::*  *NUP214* | *MECOM* | *BCR:: ABL1* | MR | *NPM1* | *CEBPA* | *NUP98* | Other | Diff,  AMD | Diff,  AWOM | Diff,  AWM | Diff,  AMML | Diff,  AMNL | Total |  |
| --- | --- | --- | --- | --- | --- | --- | --- | --- | --- | --- | --- | --- | --- | --- | --- | --- | --- | --- | --- | --- |
| WHO 2 0 1 6 | *RUNX1-RUNX1T1* | 69 |  |  |  |  |  |  |  |  |  |  |  |  |  |  |  |  | 69 |  |
|  | *PML*-*RARA* |  | 82 |  |  |  |  |  |  |  |  |  |  |  |  |  |  |  | 82 |  |
|  | *CBFB*-*MYH11* |  |  | 45 |  |  |  |  |  |  |  |  |  |  |  |  |  |  | 45 |  |
|  | *MLLT3*-*KMT2A* |  |  |  | 15 |  |  |  |  |  |  |  |  |  |  |  |  |  | 15 |  |
|  | *DEK*-*NUP214* |  |  |  |  | 4 |  |  |  |  |  |  |  |  |  |  |  |  | 4 |  |
|  | *GATA2*, *MECOM* |  |  |  |  |  | 9 |  |  |  |  |  |  |  |  |  |  |  | 9 |  |
|  | *BCR*-*ABL1* |  |  |  |  |  |  | 5 |  |  |  |  |  |  |  |  |  |  | 5 |  |
|  | MRC |  |  |  | 8 |  | 1 |  | 186 | 6 | 3 | 4 | 1 | 4 | 8 | 14 | 5 | 2 | 242 |  |
|  | *NPM1* |  |  |  |  |  |  |  |  | 144 |  |  |  |  |  |  |  |  | 144 |  |
|  | *CEBPA* |  |  |  |  |  |  |  |  |  | 57 |  |  |  |  |  |  |  | 57 |  |
|  | *RUNX1* |  |  |  |  |  |  |  | 20 |  | 1 |  |  | 2 | 9 | 3 | 3 | 1 | 39 |  |
|  | NOS, AMD |  |  |  | 2 |  |  |  | 5 |  |  |  |  | 7 |  |  |  |  | 14 |  |
|  | NOS, AWOM |  |  |  | 3 |  |  |  | 13 |  | 3 | 1 |  |  | 40 |  |  |  | 60 |  |
|  | NOS, AWM |  |  |  | 2 |  |  |  | 10 |  | 1 |  | 1 |  |  | 29 |  |  | 43 |  |
|  | NOS, AMML |  |  |  | 4 |  |  |  | 5 |  |  |  |  |  |  |  | 13 |  | 22 |  |
|  | NOS, AMNL |  |  |  | 4 |  |  |  | 2 |  |  | 1 |  |  |  |  |  | 2 | 9 |  |
|  | NOS, AMKL |  |  |  |  |  |  |  | 2 |  |  |  |  |  |  |  |  |  | 2 |  |
| Total | | 69 | 82 | 45 | 38 | 4 | 10 | 5 | 243 | 150 | 65 | 6 | 2 | 13 | 57 | 46 | 21 | 5 | 861 |  |
| *WHO* World Health Organization classification, *MRC* acute myeloid leukemia with myelodysplasia-related changes, *NOS* not otherwise specified, *AMD* acute myeloid leukemia with minimal differentiation, *AWOM* acute myeloid leukemia without maturation, *AWM* acute myeloid leukemia with maturation, *AMML* acute myelomonocytic leukemia, *AMNL* acute monoblastic and monocytic leukemia, *AMKL* acute megakaryoblastic leukemia, *MR* acute myeloid leukemia with myelodysplasia-related, *Other* acute myeloid leukemia with other defined genetic alterations*, Diff* acute myeloid leukemia, defined by differentiation. | | | | | | | | | | | | | | | | | | | | |

Supplementary Table2. Comparison of the distribution of acute myeloid leukemia patients between WHO2022 and ICC classification

| WHO 2 0 2 2 | ICC | *RUNX1::* *RUNX1T1* | *PML:: RARA* | *CBFB::*  *MYH11* | *MLLT3::*  *KMT2A* | Other *KMT2A* | *DEK::*  *NUP214* | *GATA2*; *MECOM* | other *MECOM* | *BCR:: ABL1* | Other Translo | *NPM1* | *CEBPA* | *TP53* | MR-M | MR-C | NOS | NOS,  Hx | Total |
| --- | --- | --- | --- | --- | --- | --- | --- | --- | --- | --- | --- | --- | --- | --- | --- | --- | --- | --- | --- |
|  | *RUNX1::RUNX1T1* | 69 |  |  |  |  |  |  |  |  |  |  |  |  |  |  |  |  | 69 |
|  | *PML*::*RARA* |  | 82 |  |  |  |  |  |  |  |  |  |  |  |  |  |  |  | 82 |
|  | *CBFB*::*MYH11* |  |  | 45 |  |  |  |  |  |  |  |  |  |  |  |  |  |  | 45 |
|  | *KMT2A* |  |  |  | 15 | 23 |  |  |  |  |  |  |  |  |  |  |  |  | 38 |
|  | *DEK*::*NUP214* |  |  |  |  |  | 4 |  |  |  |  |  |  |  |  |  |  |  | 4 |
|  | *MECOM* |  |  |  |  |  |  | 9 | 1 |  |  |  |  |  |  |  |  |  | 10 |
|  | *BCR*::*ABL1* |  |  |  |  |  |  |  |  | 5 |  |  |  |  |  |  |  |  | 5 |
|  | MR |  |  |  |  |  |  |  |  |  | 1 |  |  | 33 | 145 | 52 | 2 | 10 | 243 |
|  | *NPM1* |  |  |  |  |  |  |  |  |  | 1 | 149 |  |  |  |  |  |  | 150 |
|  | *CEBPA* |  |  |  |  |  |  |  |  |  |  |  | 57 |  | 2 |  | 6 |  | 65 |
|  | *NUP98* |  |  |  |  |  |  |  |  |  | 6 |  |  |  |  |  |  |  | 6 |
|  | Other |  |  |  |  |  |  |  |  |  | 2 |  |  |  |  |  |  |  | 2 |
|  | Diff, AMD |  |  |  |  |  |  |  |  |  |  |  |  |  | 3 | 3 | 7 |  | 13 |
|  | Diff, AWOM |  |  |  |  |  |  |  |  |  | 1 |  |  |  | 10 | 9 | 37 |  | 57 |
|  | Diff, AWM |  |  |  |  |  |  |  |  |  |  |  |  |  | 5 | 7 | 34 |  | 46 |
|  | Diff, AMML |  |  |  |  |  |  |  |  |  |  |  |  | 1 | 3 | 3 | 14 |  | 21 |
|  | Diff, AMNL |  |  |  |  |  |  |  |  |  |  |  |  |  | 1 | 1 | 3 |  | 5 |
|  | Total | 69 | 82 | 45 | 15 | 23 | 4 | 9 | 1 | 5 | 11 | 149 | 57 | 34 | 169 | 75 | 103 | 10 | 861 |

*ICC* International Consensus Classification, *WHO* World Health Organization classification, *MR* acute myeloid leukemia with myelodysplasia-related, *Other* acute myeloid leukemia with other defined genetic alterations*, Diff* acute myeloid leukemia, defined by differentiation, *AMD* acute myeloid leukemia with minimal differentiation, *AWOM* acute myeloid leukemia without maturation, *AWM* acute myeloid leukemia with maturation, *AMML* acute myelomonocytic leukemia, *AMNL* acute monocytic leukemia, *Other translo* acute myeloid leukemia with other rare recurring translocations, *MR-M* acute myeloid leukemia with myelodysplasia-related gene mutations, *MR-C* acute myeloid leukemia with myelodysplasia-related cytogenetic abnormalities, *NOS* not otherwise specified, *Hx* with history of myelodysplastic syndrome or myelodysplastic syndrome/myeloproliferative disorder.

Supplementary Table3. Comparison of the distribution of acute myeloid leukemia patients between WHO2016 and ICC classification

| WHO 2 0 1 6 | ICC | *RUNX1::* *RUNX1T1* | *PML:: RARA* | *CBFB::*  *MYH11* | *MLLT3::*  *KMT2A* | Other *KMT2A* | *DEK::*  *NUP214* | *GATA2*; *MECOM* | other *MECOM* | *BCR:: ABL1* | Other Translo | *NPM1* | *CEBPA* | *TP53* | MR-M | MR-C | NOS | NOS,  Hx | Total |
| --- | --- | --- | --- | --- | --- | --- | --- | --- | --- | --- | --- | --- | --- | --- | --- | --- | --- | --- | --- |
|  | *RUNX1-RUNX1T1* | 69 |  |  |  |  |  |  |  |  |  |  |  |  |  |  |  |  | 69 |
|  | *PML*-*RARA* |  | 82 |  |  |  |  |  |  |  |  |  |  |  |  |  |  |  | 82 |
|  | *CBFB*-*MYH11* |  |  | 45 |  |  |  |  |  |  |  |  |  |  |  |  |  |  | 45 |
|  | *MLLT3*-*KMT2A* |  |  |  | 15 |  |  |  |  |  |  |  |  |  |  |  |  |  | 15 |
|  | *DEK*-*NUP214* |  |  |  |  |  | 4 |  |  |  |  |  |  |  |  |  |  |  | 4 |
|  | *GATA2*, *MECOM* |  |  |  |  |  |  | 9 |  |  |  |  |  |  |  |  |  |  | 9 |
|  | *BCR*-*ABL1* |  |  |  |  |  |  |  |  | 5 |  |  |  |  |  |  |  |  | 5 |
|  | MRC |  |  |  |  | 8 |  |  | 1 |  | 7 | 5 | 3 | 33 | 93 | 62 | 20 | 10 | 242 |
|  | *NPM1* |  |  |  |  |  |  |  |  |  |  | 144 |  |  |  |  |  |  | 144 |
|  | *CEBPA* |  |  |  |  |  |  |  |  |  |  |  | 49 |  | 2 |  | 6 |  | 57 |
|  | *RUNX1* |  |  |  |  |  |  |  |  |  |  |  | 1 |  | 38 |  |  |  | 39 |
|  | NOS, AMD |  |  |  |  | 2 |  |  |  |  |  |  |  |  | 5 |  | 7 |  | 14 |
|  | NOS, AWOM |  |  |  |  | 3 |  |  |  |  | 1 |  | 3 |  | 13 | 8 | 32 |  | 60 |
|  | NOS, AWM |  |  |  |  | 2 |  |  |  |  | 2 |  | 1 |  | 9 | 4 | 25 |  | 43 |
|  | NOS, AMML |  |  |  |  | 4 |  |  |  |  |  |  |  | 1 | 5 | 1 | 11 |  | 22 |
|  | NOS, AMNL |  |  |  |  | 4 |  |  |  |  | 1 |  |  |  | 2 |  | 2 |  | 9 |
|  | NOS, AMKL |  |  |  |  |  |  |  |  |  |  |  |  |  | 2 |  |  |  | 2 |
|  | Total | 69 | 82 | 45 | 15 | 23 | 4 | 9 | 1 | 5 | 11 | 149 | 57 | 34 | 169 | 75 | 103 | 10 | 861 |

*ICC* International Consensus Classification, *WHO* World Health Organization classification, *MRC* acute myeloid leukemia with myelodysplasia-related changes, *AMD* acute myeloid leukemia with minimal differentiation, *AWOM* acute myeloid leukemia without maturation, *AWM* acute myeloid leukemia with maturation, *AMML* acute myelomonocytic leukemia, *AMNL* acute monoblastic and monocytic leukemia, *AMKL* acute megakaryoblastic leukemia, *Translo* gene translocation, *Other translo* acute myeloid leukemia with other rare recurring translocations, *MR-M* acute myeloid leukemia with myelodysplasia-related gene mutations, *MR-C* acute myeloid leukemia with myelodysplasia-related cytogenetic abnormalities, *Hx* with history of myelodysplastic syndrome or myelodysplastic syndrome/myeloproliferative disorder.

Supplementary Table4. Overall survival of acute myeloid leukemia subgroups.

|  | Overall survival median, months (95% CI) |
| --- | --- |
| *RUNX1*::*RUNX1T1* (n=69) | 41.3 (34.9-47.7)* |
| *PML*::*RARA* (n=82) | 53.6 (48.4-58.8)* |
| *CBFB*::*MYH11* (n=45) | 43.8 (35.8-51.7)* |
| *KMT2A*, WHO2022 (n=38) | 23.0 (12.0-25.0) |
| *DEK*::*NUP214* (n=4) | 31.8 (19.4-44.1)* |
| *MECOM*, WHO2022 (n=10) | 5.0 (2.0-22.0) |
| *BCR*::*ABL1* (n=5) | 1.0 (0.0-5.0) |
| MR, WHO2022 (n=243) | 10.0 (7.0-13.0) |
| MR with Hx, WHO2022 (n=54) | 6.0 (4.0-8.0) |
| MR without Hx, WHO2022 (n=189) | 13.0 (9.0-16.0) |
| *NPM1*, WHO2022 (n=150) | 12.0 (9.0-15.0) |
| *CEBPA*, WHO2022 (n=65) | 46.7 (40.6-52.8)* |
| *NUP98*, WHO2022 (n=6) | 9.0 (1.0-26.0) |
| Other, WHO2022 (n=2) | 10.0 (10.0-15.0) |
| Diff, WHO2022 (n=142) | 23.0 (17.0-31.0) |
| Diff, AMD, WHO2022 (n=13) | 33.0 (11.0-38.0) |
| Diff, AWOM, WHO2022 (n=57) | 21.0 (13.0-31.0) |
| Diff, AWM, WHO2022 (n=46) | 21.0 (11.0-42.0) |
| Diff, AMML, WHO2022 (n=21) | 22.0 (13.0-27.0) |
| Diff, AMNL, WHO2022 (n=5) | 27.4 (12.7-42.1)* |
| *MLLT3::KMT2A*, ICC (n=15) | 25.0 (1.0-25.0) |
| Other *KMT2A*, ICC (n=23) | 22.0 (10.0-23.0) |
| *GATA2, MECOM*, ICC (n=9) | 5.0 (2.0-40.0) |
| Other *MECOM*, ICC (n=1) | 18.0 (18.0-18.0) |
| Other Translo, ICC (n=11) | 17.0 (2.0-26.0) |
| *NPM1*, ICC (n=149) | 12.0 (9.0-15.0) |
| *CEBPA*, ICC (n=57) | 47.9 (41.4-543) |
| *TP53*, ICC (n=34) | 3.0 (2.0-6.0) |
| MR (M or C), ICC (n=244) | 13.0 (10.0-17.0) |
| MR-M, ICC (n=169) | 13.0 (8.0-16.0) |
| MR-C, ICC (n=75) | 15.0 (9.0-19.0) |
| NOS, ICC (n=103) | 22.0 (17.0-31.0) |
| NOS, Hx, ICC (n=10) | 9.0 (0.0-15.0) |
| NOS with or without Hx, ICC (n=113) | 21.0 (15.0-31.0) |

*CI* Cconfidence interval, *WHO* World Health Organization classification, *MR* acute myeloid leukemia with myelodysplasia-related, *Hx* history of myelodysplastic syndrome or myelodysplastic syndrome/myeloproliferative disorder, *Other* acute myeloid leukemia with other defined genetic alterations, *Diff* acute myeloid leukemia, defined by differentiation, *AMD* acute myeloid leukemia with minimal differentiation, *AWOM* acute myeloid leukemia without maturation, *AWM* acute myeloid leukemia with maturation, *AMML* acute myelomonocytic leukemia, *AMNL* acute monocytic leukemia, *ICC* International Consensus Classification, *Other translo* acute myeloid leukemia with other rare recurring translocations, *MR (M or C)* Acute myeloid leukemia with myelodysplasia-related gene mutations or cytogenetic abnormalities, *MR-M* acute myeloid leukemia with myelodysplasia-related gene mutations, *MR-C* acute myeloid leukemia with myelodysplasia-related cytogenetic abnormalities, *NOS* not otherwise specified.

* When the number of analysis patients was small or the overall survival was long, the median value was not calculated, so the mean (95% CI) was recorded.

Supplementary Figure1. Genetic profile of acute myeloid leukemia with myelodysplasia-related (AML-MR) patients according to WHO 2022

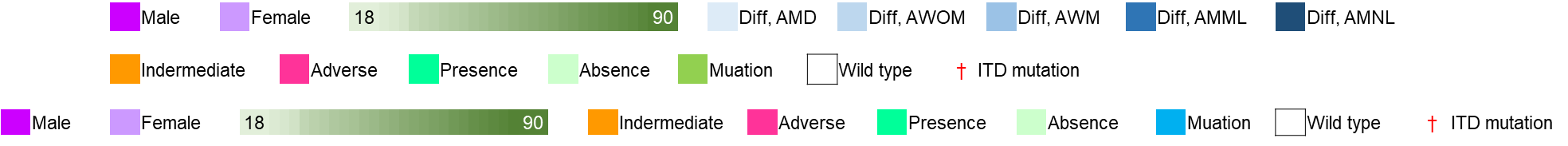


Each column represents one patient, and each row represents genetic or clinical information. Information is indicated by color and color intensity.

*ELN* European LeukemiaNet, *MR-Mutation* patients with myelodysplasia-related gene mutations. *MR-Cytogenetic* patients with myelodysplasia-related cytogenetic abnormalities. *MR-History* patients with history of MDS or MDS/MPN, *ITD* internal tandem duplication

Supplementary Figure2. Genetic profile of acute myeloid leukemia, defined by differentiation (AML, diff) patients according to WHO 2022

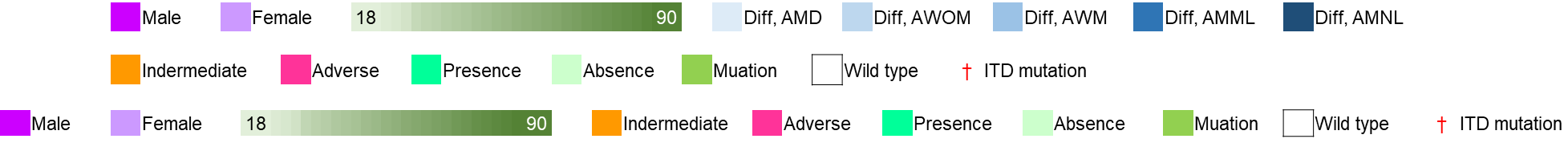


Each column represents one patient, and each row represents genetic or clinical information. Information is indicated by color and color intensity.

*ELN* European LeukemiaNet, *Diff* acute myeloid leukemia, defined by differentiation, *AMD* acute myeloid leukemia with minimal differentiation, *AWOM* acute myeloid leukemia without maturation, *AWM* acute myeloid leukemia with maturation, *AMML* acute myelomonocytic leukemia, *AMNL* acute monocytic leukemia, *ITD* internal tandem duplication

**References**

1. McGowan-Jordan J, Hastings RJ, Moore S. ISCN 2020: An International System for Human Cytogenomic Nomenclature (2020). Basel, Switzerland: Karger; 2020. 163 p.

2. Kim HJ, Kim Y, Kang D, Kim HS, Lee JM, Kim M, et al. Prognostic value of measurable residual disease monitoring by next-generation sequencing before and after allogeneic hematopoietic cell transplantation in acute myeloid leukemia. Blood Cancer J. 2021;11(6):109.

3. Li MM, Datto M, Duncavage EJ, Kulkarni S, Lindeman NI, Roy S, et al. Standards and Guidelines for the Interpretation and Reporting of Sequence Variants in Cancer: A Joint Consensus Recommendation of the Association for Molecular Pathology, American Society of Clinical Oncology, and College of American Pathologists. J Mol Diagn. 2017;19(1):4-23.

4. Kim Y, Lee GD, Park J, Yoon JH, Kim HJ, Min WS, et al. Quantitative fragment analysis of FLT3-ITD efficiently identifying poor prognostic group with high mutant allele burden or long ITD length. Blood Cancer J. 2015;5(8):e336.
